# External comparator studies and the joint application of the estimand and target trial emulation frameworks

**DOI:** 10.3389/fdsfr.2024.1409102

**Published:** 2024-10-30

**Authors:** Gerd Rippin, Héctor Sanz

**Affiliations:** ^1^ IQVIA, Biostatistics, Frankfurt, Germany; ^2^ IQVIA, Biostatistics, Barcelona, Spain

**Keywords:** external comparator studies, external control arm studies, externally controlled trials, historical control studies, estimands, target trial emulation

## Abstract

The estimand framework (EF) and the target trial emulation framework (TTEF) are two important frameworks that can inform the design and analysis of external comparator (EC) studies. The EF helps clarifying the quantity to be estimated (the “estimand”), especially regarding the handling of post-baseline intercurrent events which interfere with the existence or interpretation of endpoints. Similarly, the TTEF is instrumental for specifying key design components of a hypothetical randomized trial and identifying which of these an EC study can and cannot emulate. We offer considerations about the joint application of both frameworks by combining the five EF attributes— *treatment, population, endpoint, intercurrent events,* and *population-level summary*—with the seven TTEF components— *eligibility criteria*, *treatment strategies*, *assignment procedures*, *follow-up period*, *outcomes*, *causal contrasts*, and *data analysis plan.* Any overlap is identified, as well as omissions and unique contributions from both frameworks. Furthermore, we highlight specific considerations when applying these joint elements to EC studies.

## 1 Introduction

External comparator (EC) studies compile external comparator data for clinical trials, such as a single-arm-trial (SAT), to mitigate the lack of an internal control group and contextualize findings, potentially including formal hypothesis testing (U.S. Food and Drug Administration, 2023; Rippin et al., 2022; Burger et al., 2021; Ghadessi et al., 2020). In this setting, consideration of the two frameworks of estimands (ICH E9(R1) Expert Working Group, 2021) and target trial emulation (Hernán and Robins, 2016) is recommended, see (European Medicines Agency, 2024; U.S. Food and Drug Administration, 2024).

The target trial emulation framework (TTEF) was formally introduced by Hernán and Robins (2016) to inform the design of observational studies while also commenting on some analysis strategies. The framework guides researchers to specify key components of a hypothetical randomized clinical trial (RCT) which could answer the research question at hand. Thereafter, these components are emulated with observational data as far as possible. This procedure helps specifying “relevant causal contrasts” and prevents “selection and immortal time biases” while increasing “…the transparency and replicability of observational effect estimates” (Hernán, 2021). Technically, the TTEF consists of seven components: *eligibility criteria*, *treatment strategies*, *assignment procedures*, *follow-up period*, *outcomes*, *causal contrasts*, and *data analysis plan*. Considerations for applying the TTEF to EC studies have been described in Arnold et al. (2024).

Another important framework that can inform the conduct of EC studies is the estimand framework (EF) as defined by the International Council of Harmonisation (ICH) E9(R1) addendum, which aims to clearly define “…the treatment effect reflecting the clinical question posed by the trial objective” (ICH E9(R1) Expert Working Group, 2021). This goal is aimed to be achieved by specifying the five estimand attributes: *treatment, population, endpoint* (or *variable*), *intercurrent events* (ICEs), and *population-level summary*. General introductions to estimands (e.g., Gogtay et al., 2021) and more specific considerations for observational studies (Li et al., 2022; Chen et al., 2023; Wu et al., 2023) and EC studies (Rippin et al., 2022; Rippin, 2024) are available.

Both frameworks should be applied for EC studies and non-interventional studies in general. The European Medicines Agency (EMA) states that, “The target trial emulation (TTE) framework should be considered …”, and “…the estimand framework described in the ICH E9 (R1) Addendum on Estimands and Sensitivity Analysis in Clinical Trials should be considered …” (European Medicines Agency, 2024). Similarly, the US Food and Drug Administration (FDA) states that the chosen analytical approach should include information about the estimand, and the TTEF is also mentioned as an option to support defining causal contrasts (U.S. Food and Drug Administration, 2024). This is further supported by the Duke Margolis Institute for Health Policy White Paper, stating that “The estimand and the target trial framework can be usefully combined to determine causality…” (Duke Institute of Health Policy, 2024). Consequently, the joint application of the EF and TTEF has been performed in the context of EC studies before (e.g. Polito et al., 2024; Hampson et al., 2024).

However, there are some issues when applying both frameworks jointly.

1. The overlap of frameworks is currently handled in practical EC studies by individual solutions without cross-study standardization. As examples, see the different structures in Table 1 of Polito et al., 2024 and Table 1 of Hampson et al., 2024, indicating a lack of consistency and undesirable diversity when presenting relevant framework information.
2. The overlap in both frameworks may be seen to be inefficient and unsatisfactory.

**TABLE 1:** Unifying elements of the estimand and target trial emulation frameworks.

Unifying element #	EF attribute #	TTEF component #	Comments
1 Treatment Conditions and Strategies	1. Treatment	2. Treatment strategies	EF focuses on treatment conditions while the TTEF emphases design considerations
2 Population	2. Population	1. Eligibility criteria	The TTEF adds the point of RCT patients engaged in the trial may be approximated by EC patients having regular healthcare contacts prior to baseline (dependent on data availability)
3 Endpoint and Validation	3. Endpoint	5. Outcome	TTEF adds misclassification and validation as further points of consideration
4 Intercurrent Events (ICEs)	4. Intercurrent events	6. Causal contrasts of interest	The EF terminology is a complete guide to how to handle ICEs, while the TTEF addresses treatment-related ICEs only
5 Population-level Summary	5. Population-level summary	*Not mentioned*	Not mentioned in the TTEF
6 Follow-up Period	*Not mentioned*	4. Follow-up period	Not mentioned in the EF
7 Baseline	*Not mentioned*	7a. Analysis Plan: Defining Time Zero 7b. Analysis Plan: Specifying a Grace Period for Treatment Initiation	Typically trivial for trials, so missing in the EF
8 Assignment Procedures	*Not mentioned*	3. Assignment procedures	Specifies whether the study is randomized . If not, includes a data quality assessment regarding the ability to emulate randomization Randomization is discussed in the EF but does not constitute a formal EF attribute
9 Marginal Estimator	*Not mentioned*	*Not mentioned*	Trivial for trials, so missing in the EF. This is not mentioned in the TTEF as well

Note that the elements in the same rows do not have to be identical but can be considered to have sufficient overlap to form a basis for a new unifying element.

Based on these issues, this research publication offers considerations about:

1. developing a standardized approach for a systematic presentation of the joint application of both frameworks. This is equivalent to proposing a tangible set of unifying framework elements with clear rationale and terminology.
2. exploring whether the two frameworks can be merged, and developing an understanding about any challenges.

The first research goal is addressed by systematically describing the unique contributions and overlap of both frameworks and any omissions to derive mutually exclusive unifying elements (Sections 2.1–2.9). Based on this, Section 3 addresses the question of joining frameworks and related discussions.

## 2 Relationships between the EF and TTE frameworks

Both frameworks consist of different but partially overlapping elements. In case of overlap for a specific element, the frameworks may still have unique contributions and perspectives. However, there are also elements which are unique to either. Table 1 shows a list of unifying elements of EF attributes and TTEF components, further considerations, and short summary notes. Each table item is described in the corresponding Sections 2.1–2.9 in detail.

### 2.1 EF attribute #1 *treatment* and TTEF component #2 *treatment strategies*: unifying element #1 *treatment conditions and strategies*

There is a rationale for joining the EF attribute *Treatment* and the TTEF component *Treatment Strategies* due to overlap to derive unifying element #1 *Treatment Conditions and Strategies*. This describes all the details regarding the treatment (including route of administration, frequency, dose, and planned post-baseline treatment patterns).

The TTEF clarifies that no placebo comparison can be performed in a purely observational setting. For EC studies, however, it may be that a previous RCT placebo arm is available as a comparator. Other design considerations are also mentioned in the TTEF. For example, applying a “new-user design,” which prevents biases associated with eligibility criteria being “…defined after the initiation of a treatment strategy and therefore are possibly affected by the strategy itself” (Hernán and Robins, 2016).

While these TTEF considerations are relevant for EC studies, they are naturally not reflected in the EF due to the intentional focus on clinical trials. A clinical trial design based on new users is common due to the experimental treatment being new and not yet approved for the indication. Furthermore, there is a clear time point from which patients are followed prospectively, and no retrospective design is applied.

For EC studies, the application of the TTEF is strongly supported by a (draft) SAT protocol being already available because it can be directly leveraged to mimic the SAT design in the EC cohort to the best possible extent. Hence, the emulation task is simplified for EC studies if the SAT has been designed already. If not, the emulation of a hypothetical RCT can be further optimized, for example, by including in the SAT real-world endpoints which are measured in observational data sources and which would not have been considered if the SAT was planned without an EC cohort.

Note that unifying element #1 is not intended to cover any analytical handling of dynamic treatment strategies and post-baseline exposure events, as this is a statistical analysis topic which is well-covered by EF attribute #4 on deriving estimands when considering intercurrent events (see unifying element #4). In this sense, a part of TTEF component #2 is assigned to a different unifying element.

### 2.2 EF attribute #2 *population* and TTEF component #1 *eligibility criteria*: unifying element #2 *population*

There is substantial overlap in the two frameworks regarding EF attribute #2 *Population* and TTEF component #1 *Eligibility Criteria*. While the population can be defined in a clinical trial in a very granular way by customized screening measurements, the TTEF phrasing of *Eligibility Criteria* is more modest, potentially because sometimes it is not the desired population itself that is described but rather its approximation by those eligibility criteria which are available in an observational data source. For EC studies, it is important to discuss differences in the cohorts’ eligibility criteria, as these essentially lead to two different sets of populations, and the extent of population differences across cohorts is an important factor when interpreting EC results. Also, it is important at the early stage of study feasibility to check whether the EC can be made sufficiently similar to the SAT by means of available data variables.

The TTEF adds the point that, when using healthcare data, an emulated trial includes patients who “…are expected to remain actively engaged” (Hernán and Robins, 2016). This could be mimicked in the EC by, for example, restricting “…the analysis to individuals who have been in regular contact with the healthcare system before baseline” (Hernán and Robins, 2016). However, this depends on the availability of such information in the selected data source and whether the sample size is still sufficient after applying such a data restriction; this may not be the case for rare disease EC studies.

### 2.3 EF attribute #3 *endpoint* and TTEF component #5 *outcome*: unifying element #3 *endpoint and validation*

Again, there is high overlap in the two frameworks regarding EF attribute #3 *endpoint* and TTEF component #5 *Outcome*, suggesting that unifying element #3 should be named *Endpoint and Validation*. The TTEF adds the important topic of outcome misclassification and validation approaches, which is a relevant topic for EC studies, where the validity of observational endpoints and their comparability with trial data must be discussed thoroughly.

The EF, however, does not address outcome misclassification and validation approaches. It can be argued that endpoint validation does correct the estimate on the estimator level and does not relate to the estimand level. Still, there is an option to reflect differences in the accuracy of endpoints across cohorts on the estimand level by using different names for trial and EC endpoints. For example, the endpoint name for the EC endpoint could include a prefix like “*real-world* progression-free survival” (rwPFS), while no such prefix is applied for the trial data (PFS). Such an approach would transparently indicate a potential difference in measurement accuracy on the estimand level.

Note that the term *Endpoint* always includes the specification of the timepoint or time period of measurement. This follows from the ICH E9(R1) addendum, which states that “An estimand is a precise description of the treatment effect …” (ICH E9(R1) Expert Working Group, 2021). This precision can only be reached if the timepoint or time frame of the endpoint is specified. Examples of endpoint definitions are “change in systolic blood pressure after 3 months” or “overall survival during a follow-up period of 3 years.” See also Section 2.6 for a related discussion about the study’s follow-up period.

### 2.4 EF attribute #4 *intercurrent events* and TTEF component #5 *causal contrasts of interests*: unifying element #4 *intercurrent events*

EF attribute #4 about *Intercurrent Events* is the core element of the estimand framework. It offers a thorough and sophisticated concept for handling all kinds of intercurrent events; these are not necessarily restricted to treatment-related intercurrent events (as discussed in the TTEF). The discussion in the TTEF is also limited by intention-to-treat (ITT) and per-protocol (PP) considerations, so that it is suggested to keep the name *Intercurrent Events* for a new unifying element.

The classic clinical trial approach of assessing treatments is based on the intention-to-treat population, which is usually not available in EC studies because it is typically not recorded which patients were intended to be treated but just which patients have actually received treatment. There are exceptions, though, as there are observational data sources which are based on prescribed treatments, where defining an ITT population may be possible. These data sources, however, may not be optimal for EC studies due to a potential lack of granular information regarding important prognostic factors.

Notably, an ITT population can be analyzed by five different estimand strategies (ICH E9(R1) Expert Working Group, 2021) that estimate conceptually very different quantities, which is not addressed in the TTEF. The estimand treatment policy is one of these five strategies and does not adjust for any intercurrent event, including those which are related to treatment exposure, as “the intercurrent event is considered to be part of the treatments being compared” (ICH E9(R1) Expert Working Group, 2021). The treatment policy strategy is often requested by regulatory stakeholders. An alternative estimand strategy is the hypothetical estimand, which can be constructed by modeling the scenario in which an intercurrent event would (or would not) have occurred. Such a strategy can be applied, for example, for per-protocol analyses, treatment switching, subsequent therapies, or for events inducing informative censoring in general.

It is important to understand that there is no unique ITT analysis according to the EF, so that the TTEF usage of this term is ambiguous from the EF perspective when not specifying which estimand strategy is chosen. Hence, it would be a misunderstanding to think that an ITT analysis would always imply the treatment policy estimand. One counter example is provided in the EF, where it is stated that “…the treatment policy strategy cannot be implemented for intercurrent events that are terminal events” (ICH E9(R1) Expert Working Group, 2021). However, in such a setting, a composite endpoint estimand strategy may be able to provide a meaningful ITT analysis approach. One example for a composite endpoint is PFS, where death as an intercurrent event is incorporated into the endpoint itself, such that progression and death are both counted as events.

Causal contrasts in terms of different marginal estimators such as the average treatment effect (ATE), the average treatment effect on the treated (ATT), the average treatment effect on the untreated (ATU), and the average treatment effect in the overlap population (ATO) are not discussed in the EF or TTEF but are suggested to be added as per element #9 (Section 2.9).

### 2.5 EF attribute #5 *population-level summary*: unifying element #5 *population-level summary*

This EF attribute has no counterpart in the TTEF and can be mapped 1:1 to unifying element #5: *Population-level summary*. It was noted before that not all population-level summaries can be recommended for EC studies (Rippin, 2024), so that its specification needs careful consideration. For example, the hazard ratio may not be the best population-level summary because the underlying assumption of proportional hazards is likely to become fragile when mixing different data sources.

### 2.6 TTEF component #4 *follow-up period*: unifying element #6 *follow-up period*

The follow-up period is not mentioned in the EF and can be mapped 1:1 to the unifying element #6 *Follow-up Period*.

One practical example of why it can be useful to have this element in place is a treatment effect which has been observed during a rather short follow-up time which could have diminished later. For example, a short-term improvement in patients’ reported outcomes or ejection fractions for coronary diseases may not lead to a long-term superiority of one treatment over another.

The follow-up time is considered a characteristic of the trial design, which may not necessarily map 1:1 to the timing of all planned endpoints. For example, in a 2-year study, one endpoint could be a 6 minutes walking distance (6MWD) test after 12 months, while another could be a 6MWD after 24 months.

However, the EF is acting on the endpoint level, not the study design level, such that the follow-up period does not have a natural place in the EF (see also Section 2.3). Hence, the follow-up time is only needed in the unifying framework which also describes the study design level.

### 2.7 TTEF component #7 *analysis plan – defining time zero* and *specifying a grace period*: unifying element #7 *baseline*

Defining baseline is not mentioned in the EF because there are rarely issues in clinical trials where the randomization date is typically taken for RCTs and enrollment or treatment start dates for SATs. However, for EC studies baseline was suggested to be added as an additional estimand attribute before (Rippin, 2024), and the TTEF also discusses this topic in detail. For example, multiple baseline time points for the same patient could occur, for example, by a patient being eligible when starting a third, fourth, or higher line of treatment, which can be leveraged in EC studies to increase sample size, which may become especially important for applications in the area of rare diseases.

The TTEF also mentions the possibility of specifying a grace period to initiate treatment after assignment, which would need correct statistical handling to avoid bias. On the other hand, taking treatment initiation as index date for an EC study will lead to simplifications of the statistical analysis.

### 2.8 TTEF component #3 *assignment procedures*: unifying element #8 *assignment procedures*

TTEF component #3 *Assignment Procedures* specifies whether a study is randomized. For EC studies, however, it is clear that the assignment procedure is not based on randomization. In such a case, the data quality of the covariates needs to be sufficient to conceptualize the research as a “conditional RCT” based on the patients’ baseline prognostic factors (Hernán and Robins, 2021), where randomization can be emulated by means of statistical adjustment. Hence, an assessment of associated data quality is implicitly included when discussing emulating randomization for observational research settings.

Granular sub-categories of data quality may be defined, including missingness and accuracy considerations (mismeasurement and misclassification), and whether the data is generally fit for purpose (Levenson et al., 2023; Chen et al., 2023). The topic of adequate data quality (reliability and relevance) is also very prominent in important regulatory documents (European Medicines Agency, 2023; European Medicines Agency, 2024; U.S. Food and Drug Administration, 2024).

The EF, on the other hand, does not discuss data quality. This may be because it can be argued that data quality is related to the quality of the estimate but not to the quantity aimed to be estimated (the estimand). As such, it would not have a natural place in the EF. Still, the likelihood and magnitude of bias are important to understand for interpreting the results generated for an estimand (see also Section 3).

Reduced data quality may not only occur regarding covariates but for all underlying data associated with unifying elements #1–#4. A discussion of the quality of endpoint data was assigned to element #3 due to the TTEF specifically mentioning validation. It is consistent to suggest the same approach for elements #1, #2, and #4 and to reserve element #8 for discussing covariate data quality only. However, it is also possible to argue for an assignment of all data quality assessments to a single element. If so, we suggest that an entirely new element, *Data Quality,* is created in addition.

### 2.9 EF further consideration #9 *the marginal estimator*: new element #9 *the marginal estimator*

Specifying the marginal estimator (ATE, ATT, ATU, or ATO) is not addressed in both frameworks, but was suggested previously as an additional estimand attribute because it affects the estimand conceptually (Rippin, 2024). The ATE standardizes the treatment effect according to the overall baseline distribution of prognostic characteristics (SAT plus EC) while the ATT and ATU adjust the treatment effect according to the SAT and EC baseline distributions, respectively. The ATO derives a marginal estimator by comparing patients who are in clinical equipoise (Li, 2018).

Although the ATE is estimated in an RCT in general cases (van Amsterdam and Ranganath, 2023), it is possible to argue that the ATE does not represent a natural quantity for EC studies as it does not form “a realistic target population” (Arnold et al., 2024). Results are somewhat artificial as they are directly dependent on the sample size ratio of the cohorts involved, and these are not identical due to applying a different granularity of eligibility criteria. However, if there are only minor or maybe moderate differences between cohorts, the ATE could be justified to approximate a meaningful quantity. In addition, for some analysis strategies, ATE-weighting may be beneficial for bias reduction in estimates (Rippin et al, 2024), which may even be more true for ATU-weighting as ongoing research is suggesting. Notably, the National Institute for Health and Care Excellence (NICE) has declared the ATE to be of interest for observational research in general (Faria et al., 2015), but not concretely for the EC study design.

A preference for the ATT would be in line with the argument that the EC aims to mimic the counterfactual by replacing the missing internal SAT control group. Moreover, it is an advantage that it can maintain the original trial results in the typical case, such that the EC provides additional information but does not change original SAT results (Rippin et al., 2022).

The ATU is also well-justifiable and interpretable because it estimates the treatment effect which is relevant for the comparator population. This perspective could be useful for HTAs (Arnold et al., 2024).

The ATO specifies a marginal estimator which is clinically relevant because the EC study is designed to focus on patients in clinical equipoise. It has a different method of interpretation than the traditional marginal estimators ATE, ATT, and ATU, which may lead to an assessment of less direct interpretability although being clinically relevant (Li et al., 2022). The emphasis on internal validity and on “…the (sub)population closest to the population in a randomized clinical trial…” (Li, 2018) does constitute a helpful perspective that other marginal estimators cannot provide.

Regulatory or HTA stakeholder input should be sought before finalizing the EC study protocol to ensure that external preferences or requirements are met, and more than one marginal estimator could be specified as supplementary analyses.

## 3 Discussion

Both the EF and TTEF are instrumental for EC studies to break down the complexity of the study approach. The EF is a framework that focuses on estimable quantities and various analysis strategies for endpoints. It is essentially a statistical framework but also intends “…to strengthen the dialogue between disciplines” (ICH E9(R1) Expert Working Group, 2021). The TTEF, on the other hand, focusses mostly (but not exclusively) on study design.

While the EF excels in clarifying how estimable quantities can be handled by identifying five different ways to address intercurrent events, it does exhibit shortcomings for observational study features in general (Chen et al., 2023) and EC study features in particular (Rippin, 2024). The TTEF, on the other hand, has strengths in decomposing complex observational study design considerations to multiple framework components but has limitations in describing all possible ways of handling intercurrent events.

As a result of the complementary strengths of the two frameworks, both should be applied for EC studies in parallel (Polito et al., 2024; Hampson et al., 2024) as indicated in important regulatory documents (European Medicines Agency, 2024; U.S. Food and Drug Administration, 2024).

This joint application should happen in a structured and standardized manner, which does not seem to be available currently. Instead, diverse solutions have been implemented on the individual study level. For example, Table 1 in Polito et al. (2024) joins TTEF components and EF attributes side by side for a practical study. The element of the marginal estimator is understandably not mentioned in their Table 1 because it is not part of either framework, although it is arguably a missing element in both frameworks. Another difference to our proposal is that the topics of baseline and follow-up period were merged to “Start/end of follow-up.” Although this may be adequate for enhancing clarity in a practical study, it is not the best solution from the theoretical perspective taken by our approach, which aims to create mutually exclusive unifying elements in the most systematic manner. By keeping the two elements #6 *Follow-up Period* and #7 *Baseline* separate, it also becomes much clearer how an updated EF that meets the needs for observational research could look (see below). Table 1 of Hampson et al. (2024) also list EF and TTEF elements for a case study, but differently again, and it is likely that undesirable diversity in structure and format will increase for future ECs. However, by deriving unifying elements, we have enabled upcoming EC studies to follow a standardized structure (research goal #1 as per Introduction) when applying both frameworks in parallel.

Research goal #1 as listed in the Introduction was thus successfully accomplished by this publication. However, our proposal can only be seen as a first step to initiating a thorough scientific discussion, and we welcome further contributions that debate how to structure the joint application of the two overlapping frameworks of estimands and target trial emulation in the best possible way.

Our solution of identified unifying elements is instrumental to answer research goal #2 of whether both frameworks could be combined into a single framework. We believe that there are no principal issues in doing so due to the fact that a systematic derivation of mutually exclusive framework elements was seen to be possible. However, strong stakeholder input would be needed, including from regulatory agencies, to endorse such a joint framework; such an endeavor may thus constitute a major and long-term task.

A more modest goal could be to join the two frameworks on the pure statistical analysis level (excluding any design considerations, which the TTEF will remain to cover well). This goal could be reached by extending the EF to also reflect observational study needs. Concretely, the EF can be extended by adding the described observational study analysis (but not design) considerations of unifying elements #1–#4 according to Table 1 and Sections 2.1–2.4. Unifying element #5 *Population-level Summary* would stay as it is, while the unifying element #6 *Follow-up time* is not needed because it is a feature of the study design but not necessarily of an estimand. Adding elements #7 *Baseline* and #9 *Marginal Estimator* has been proposed before (Rippin, 2024).

At first glance, unifying element #8 *Assignment Procedures* is rather a study design feature, and it can be argued that it does not qualify as an estimand attribute because the assignment procedure and associated data quality to emulate randomization are not related to the estimand but to the bias of the estimate. However, a new estimand attribute like *Estimand Quality* based on the data quality considerations of the unifying element #8 (Section 2.8) could be helpful in case the EF is amended to account for observational study features. Further discussion about this potential EF attribute is recommended to gather multiple perspectives, especially from regulatory stakeholders.

In summary, EC studies benefit from following a highly standardized scientific approach due to the complexity of the design. Because of this, both the EF and TTEF should be applied when designing and analyzing an EC study (European Medicines Agency, 2024; U.S. Food and Drug Administration, 2024). However, jointly applying and presenting both overlapping frameworks is not yet based on an agreed structure or a theoretical discussion, which leads to undesirable and likely increasing diversity of how this practically occurs on the individual study level. Hence, we have offered considerations for a systematic and standardized approach. The successful derivation of mutually exclusive unifying elements suggests that merging the two frameworks should be possible in principle. Similarly, it was shown that updating the EF to meet observational study needs is also conceivable.

## Data Availability

The original contributions presented in the study are included in the article/supplementary material; further inquiries can be directed to the corresponding author.
